# Modes of *Escherichia coli* Dps Interaction with DNA as Revealed by Atomic Force Microscopy

**DOI:** 10.1371/journal.pone.0126504

**Published:** 2015-05-15

**Authors:** Vladislav V. Melekhov, Uliana S. Shvyreva, Alexander A. Timchenko, Maria N. Tutukina, Elena V. Preobrazhenskaya, Diana V. Burkova, Valiriy G. Artiukhov, Olga N. Ozoline, Sergey S. Antipov

**Affiliations:** 1 Department of Cell Biology, Pushchino State Institute of Natural Sciences, Pushchino, Moscow Region, Russian Federation; 2 Laboratory of New Methods in Biology, Institute for Biological Instrumentation, Russian Academy of Sciences, Pushchino, Moscow Region, Russian Federation; 3 Department of Functional Genomics and Cellular Stress, Institute of Cell Biophysics, Russian Academy of Sciences, Pushchino, Moscow Region, Russian Federation; 4 Department of Physics of Nucleoproteids, Institute of Protein Research, Russian Academy of Sciences, Pushchino, Moscow Region, Russian Federation; 5 Department of biophysics and biotechnology, Voronezh State University, Voronezh, Russian Federation; Indian Institute of Science, INDIA

## Abstract

Multifunctional protein Dps plays an important role in iron assimilation and a crucial role in bacterial genome packaging. Its monomers form dodecameric spherical particles accumulating ~400 molecules of oxidized iron ions within the protein cavity and applying a flexible N-terminal ends of each subunit for interaction with DNA. Deposition of iron is a well-studied process by which cells remove toxic Fe^2+^ ions from the genetic material and store them in an easily accessible form. However, the mode of interaction with linear DNA remained mysterious and binary complexes with Dps have not been characterized so far. It is widely believed that Dps binds DNA without any sequence or structural preferences but several lines of evidence have demonstrated its ability to differentiate gene expression, which assumes certain specificity. Here we show that Dps has a different affinity for the two DNA fragments taken from the *dps* gene regulatory region. We found by atomic force microscopy that Dps predominantly occupies thermodynamically unstable ends of linear double-stranded DNA fragments and has high affinity to the central part of the branched DNA molecule self-assembled from three single-stranded oligonucleotides. It was proposed that Dps prefers binding to those regions in DNA that provide more contact pads for the triad of its DNA-binding bundle associated with one vertex of the protein globule. To our knowledge, this is the first study revealed the nucleoid protein with an affinity to branched DNA typical for genomic regions with direct and inverted repeats. As a ubiquitous feature of bacterial and eukaryotic genomes, such structural elements should be of particular care, but the protein system evolutionarily adapted for this function is not yet known, and we suggest Dps as a putative component of this system.

## Introduction

All living organisms use specific structural proteins in order to maintain their genomes in a functional state and to protect them from damage by a variety of physical, chemical and environmental factors. In eukaryotes, the primary responsibility for implementing the functionality in safe conditions rests on five positively charged histone proteins that condense or relax particular genomic loci by interacting with DNA without sequence specificity. In prokaryotes, this function is performed by 10–12 highly abundant proteins [1–3], which interact with DNA by recognizing structural peculiarities in double helix or even bind to the specific sequence motifs in bacterial chromosome.

A total of approximately 170,000 molecules of different proteins take care about the structure of *E*.*coli* nucleoid during the exponential growth, while transition to a steady state is accompanied by an increase in their number up to ~290,000 [1]. In rapidly growing cells the most abundant nucleoid protein is Fis (**F**actor of **i**nversion **s**timulation), which number reaches 60,000 molecules per cell. In starved cells the intracellular level of Fis drops down, while the dominant protein becomes Dps (**D**NA-binding **p**rotein of **s**tarved cells, 180,000 molecules per cell) [1]. Fis and at least four other structuring proteins (IHF, Lrp, H-NS and its paralog StpA) recognize sites for which a consensus motif may be deduced [1, 4–7]. Two other nucleoid proteins (CbpA and CbpB), as well as H-NS and StpA, bind curved DNA [8–10]; while HU (**H**eat **u**nstable protein) can form complexes with a wide spectrum of different genomic loci, including bent, disordered, nicked or cruciform DNA [11–13]. Information about the interaction of Dps with DNA is less certain. It is believed that it forms only non-specific complexes with negatively charged sugar-phosphate backbone [1, 14–16].

Most architectural proteins of bacterial nucleoid operate as homo- or heterodimers (Fis, HU, CbpA, IHF, H-NS and StpA). DnaA and CbpB(Rob) function as monomers, while Lrp and Dps can form large oligomeric particles. In this pair Lrp (**L**eucine-responsive **r**egulatory **p**rotein) exists as a mixture of dimers, octamers and hexadecamers, which equilibrium depends on the presence of leucine favoring octamer configuration. The DNA segment, containing Lrp-binding site, wraps around this octamer, forming a nucleosome-like structure [17]. Dominant oligomeric form of Dps is dodecamer, which assembles from dimers [18] or trimers [19]. Dodecamers tightly bind to DNA, but the ability of smaller oligomers to form similar complexes has not been well documented yet.

Two proteins that interact with specific sequences in the DNA (Fis and Lrp) have classical helix-turn-helix DNA-binding domains [20, 21]; while most nucleoid proteins rely on “indirect readout”, i.e. employ different structural modules so as to recognize their binding sites depending on sequence-mediated conformational features [22–24]. In Dps of *E*.*coli* this function is primarily ascribed to the flexible N-terminal tails [16], containing three lysine residues at positions 5, 8 and 10, and the arginine residue at position 18. Deletion of the first 8 or 18 amino acids dramatically decreased ability of Dps to bind DNA and to aggregate with other Dps molecules [15], but X-ray analysis did not reveal typical DNA-binding modules in the N-terminal ends or any other segments on the protein surface [16]. Thus the striking affinity of *E*. *coli* Dps to DNA is currently understood as a strong electrostatic interaction between positively charged side chains of the flexible protein modules and negatively charged DNA backbone.

An absence of N-termini (as in MsDps1 of *Mycobacterium smegmatis* [18, 19]) or their decreased flexibility (as in MsDps1 of *M*. *smegmatis* [18], Dps of *Agrobacterium tumefaciens* [25] and DpsA/DpsB molecules of *Lactococcus lactis* [26]) changed the mode of interaction or decreased DNA binding/condensation capacity [27]. Thus, in Dps of *A*. *tumefaciens* N-termini are immobilized on the dodecamer surface by the net of hydrogen bonds and salt bridges, causing the inhibition of their ability to bind DNA [25]. In both *L*. *lactis* Dps proteins N-termini form short α helices [26] exposed on the surface of the protein and bind the adjacent subunits via salt bridges and hydrogen bonds. These α helices stabilize the dodecamer structure of the protein and also participate in interaction with DNA, since removal of the first 20 amino acids from DpsA impaired DNA-binding ability. But, replacement of three Lys at positions 9, 15 and 16 by Glu had no effect on interaction with DNA [26], probably because these residues in the structure of α-helices “either face, or lie parallel” to the dodecamer surface [26]. Dps homolog of *Helicobacter pylori* (HP-NAP) does not contain a positively charged N-terminus, but instead has a positively charged protein surface that directly interacts with DNA molecules [28]. So, the mode of Dps-DNA binding may differ for the Dps molecules from different bacteria but electrostatic interactions most probably play a crucial role in these processes.

There are four theoretical models that describe the *E*.*coli* Dps-DNA interaction at the sub-molecular level. They are based on the data acquired by electron [14, 26, 29–32] or atomic force [15, 18, 28] microscopy at limited resolution. The first one was suggested by Almiron et al. [14] to explain electron micrographs of the *E*.*coli* Dps:DNA complexes, being observed as a highly ordered two dimensional honeycomb-like arrays [14, 29]. The model assumes the formation of two connected hexameric rings of the Dps monomers around the DNA double helix. However, DNA-induced conformational rearrangement of spherical dodecameric particles or assembling of hexameric rings from dimers or trimers has not yet been registered. The second model was based on the observation of large Dps-DNA co-crystals [30, 31]. It assumes ability of Dps to form stacked alternating layers, within which DNA is sequestered in hollows between adjacent dodecamers. Such co-crystals were found in starved cells containing huge amount of Dps [30] and are crucial for protection of DNA from a variety of damaging agents. However, in exponentially growing cells Dps was found as a protein uniformly dispersed within the entire nucleoid [33].

The third model considered the fact that at physiological pH values both outer and internal surfaces of the Dps globule are charged negatively (IP = 6.01). So, the protein has to repel rather than to attract DNA. Since the presence of EDTA inhibited the DNA binding, it was suggested that this interaction is mediated by the bridges formed by metal ions between negatively charged protein surface and the DNA backbone [30–32, 34, 35]. Although this model does not account for functionality of positively charged N-termini, it probably can be regarded as the most versatile because several lines of evidence indicate dependence of Dps-DNA complex formation on Mg^2+^ [30–32, 34] or Fe^2+^ [35]. Finally, the model suggested by K. Zeth [36] highlights the ability of Dps for non-specific DNA binding and assumes DNA winding around the roundish dodecamer particle in a histone-like manner.

Nucleoid proteins with sequence or structural specificity participate in differential gene regulation [37]. Such information for Dps is largely lacking. For all that, it was documented that the *dps*-null mutant of *E*. *coli* had significant changes in the protein profile [14], and microarray analysis performed for the *dps*-null mutant of *S*.*enteritidis* [38] revealed hundreds of genes with *dps*-dependent transcription. We also found that deletion of *dps* affected transcription of certain genes, leaving expression of others genomic loci of *E*.*coli* unchanged [39]. All these facts assume some specificity in Dps-DNA binding, and here we tried to shed light on this problem by three different approaches using DNA fragments of different length, sequence and structural organization. Our aim was reinforced by the fact that Dps is a unique protein in the family of structuring factors providing not only physical but also chemical protection against damaging agents. Highly conserved ferroxidase center of Dps affords sequestration of toxic Fe^2+^ ions thus avoiding hydroxyl radical production through Fenton chemistry [40]. Oxidized iron ions are then stored inside the protein cavity and can be released upon reduction. This cavity can accommodate over 400 iron oxides and can hold oxides of other metals, which modify the ferromagnetic properties of mineralized core. So, Dps is now considered as a highly prospective biomolecule for nanoelectronics giving an opportunity to create nanodevices with calibrated ferromagnetic particles [41]. Some specificity in its mode of interaction with DNA can be highly favorable in designing new materials with predictable disposition of these particles.

## Materials and Methods

### Purification of Dps

The *E*.*coli* gene *dps* was amplified with primers TAATTTCTAGAACATAACATCAAGAGG and AGCTCTAGATTTATTCGATGTTAG and cloned into the expression vector pGEMΔXba [42] using the Xba I site. The nucleotide sequence of the recombinant gene, which was not modified by any tag, was checked by direct sequencing. Gene expression was carried out in *E*.*coli* BL21 (DE3) cells grown in LB Medium in the presence of ampicillin (100 μg/mL) at 37°C. Transcription of the recombinant gene was induced by 0.02 mM IPTG at OD600 0.4–0.6 and accumulation of the protein was allowed for 12 h. It was purified using ion exchange chromatography (DEAE-Sephadex A-25, GE Healthcare, Sweden) and gel-filtration on Sephadex G-200 (Pharmacia, Sweden). The protein was concentrated on Vivaspin 20 columns (Sartorius, Germany), dialyzed against the storage buffer containing 50 mM Tris-HCl (pH 8.0), 50 mM NaCl, 0.1 mM EDTA, 5% of glycerol and stored in the freezer. The final purity of the native protein was higher than 95%.

### Preparation of DNA fragments

Linear DNA fragments were obtained by PCR using the genomic DNA of *E*.*coli* K12 MG1655 and primers (Evrogen, Russia) listed in the Table 1. After PCR, these DNA fragments were purified from substrates and primers by 5% PAAG electrophoresis, extracted and dissolved in milliQ water. Concentration of the DNA fragments was determined using the spectrophotometer ND-1000 (NanoDrop Technologies Inc., USA).

**Table 1:** Primers and oligonucleotides.

	*Sequence*
***Primers***	
*dps_F1*	5’-GGAAGATCTTCCTCGGAGAAACACT-3’
*dps_R1*	5’-ATATCTAGATATATAAAGACGGTGTA-3’
*dps_F2*	5’-ATGCAGATCTTCTCGCTACTTTTC-3’
*dps_R2*	5’-TCCTCTAGATGTTATGTCCCAGT-3’
***Oligos***	
*Y1*	5'-AAAAAAAAAAAAAAAAAAAAAAAAACCCCCCCCCCCCCCCCCCCCCCCCCCCCCCCC-3'
*Y2*	5'-CCCCCCCCCCCCCCCCCCCCCCCCCCCCCCCCTTTTTTTTTTTTTTTTTTTTTTTTT-3'
*Y3*	5'-GGGGGGGGGGGGGGGGGGGGGGGGGGGGGGGGGGGGGGGGGGGGGGGGGGGGGGGGGGGGGGGG-3'
*Y5*	5’-CTTTTCCTCTACACCGTCTTTATATATCGAATTAAGAAGTCGCAATGAGTATTACTTTGTAAAT-3’
*Y6*	5’-CAAGGGTAAACGAACCTTGCGCTTTCTTAAATATTCGATATATAAAGACGGTGTAGAGGAAAAG-3’
*Y7*	5’-ATTTACAAAGTAATACTCATTGCGACTTCTTAATTTAAGAAAGCGCAAGGTTCGTTTACCCTTG-3’
*Y8*	5’-ATTTACAAAGTAATACTCATTGCGACTTCTTAATTTAAGAAAGCGCAAGGAACGTTTACCCTTG-3’
*Y9*	5’-GCATAACCATGCAGAATTTCTCGCTACTTTTCCTCTACACCGTCTTTATATATCGAATTAAGAAGTCGCAATGAGT ATTACTTTGTAAAT-3’
*Y10*	5’-CAAGGGTAAACGAACCTTGCGCTTTCTTAAATATTCGATATATAAAGACGGTGTAGAGGAAAAGTAGCGAGAAATT CTGCATGGTTATGC-3’

Branched DNA molecules were formed of two or three single-stranded oligonucleotides (Table 1), designed in such a way that each half of one fragment was complementary to the halves of one or two other fragments (Table 1). Oligonucleotides were dissolved in 5mM MgCl_2_ solution, melted separately at 97°C for 5 min, mixed and further incubated at 97°C for 5 min. Then the mixture was transferred to 70°C water bath for 10 minutes and allowed to cool gradually to room temperature (4–5 hours).

### Electrophoretic mobility shift assays

DNA-Dps complexes were prepared by mixing the DNA fragments with Dps in different molar ratios in 10 μl of buffer that contained 50 mM Tris-HCl (pH 7.5), 0.1 mM EDTA and 50 mM NaCl. Complex formation was allowed for 30 minutes at 37°C. Efficiency of binding was assessed by gel-shift assays as described in [43]. Electrophoretic fractionation was carried out in 5% PAAG in TBE buffer (89 мМ Tris-HCl, 89 мМ Boric acid, 2 мМ EDTA, pH 8.0) at 200–250 V and 70–110 mA. DNA bands were stained with ethidium bromide or AgNO_3_.

### DNAse I footprinting

The 5’-ends of primers *dps*_F1 and *dps*_R2 (Table 1) were ^32^P-labelled using T4 polynucleotide kinase (Thermo Scientific, Lithuania) and protocol of manufacturer. Three DNA fragments were PCR amplified with primer pairs ^32^P-*dps*_F1—*dp*s_R1, *dps*_F1 - ^32^P-*dp*s_R2 and *dps*_F2 - ^32^P-*dps*_R2. The amplified fragments were extracted from the gel as described in [44]. Prior the complex formation, the DNA samples (1 pmol per reaction) were incubated for 10 minutes at 37°C in 30 μl of transcription buffer, containing 50 mM Tris-HCl (pH 8.0), 0.1 mM EDTA, 0.1 mM DTT, 10 mM MgCl_2_, 50 mM NaCl and 5 mg/ml BSA (Sigma, USA). Then, the Dps protein was added in 2–10 fold molar excess and interaction was allowed for 40 minutes at 37°C. Samples were then treated with 1 μg/ml of DNAse I for 2 minutes. Cleavage was terminated by adding 35 μl of 8M ammonium acetate. The products of DNAse 1 digestion were precipitated with ethanol, dissolved in formamide buffer [44] and loaded on a 6% denaturing polyacrylamide gel. Samples were fractionated in TBE buffer and visualized by radioautography. Gels were calibrated by the ladder of G-sequencing.

### Sample preparation for atomic force microscopy (AFM)

Stored solutions of purified Dps were passed through a Sephadex G-15 column (1x5 cm^3^) to remove any aggregated particles. Collected fractions were diluted in the buffer containing 50 mM Tris-HCl (pH 7.5) and 10 mM NaCl, to the final concentration 1 ng/μl (4.4 nM of dodecamers) and 2 μl of this solution were deposited on mica for scanning. Linear DNA fragments or Y-shape structures were dissolved in 5 mM MgCl_2_ to the concentration of 1 ng/μl (4–19 nM). The Dps complexes with different DNA fragments were formed at room temperature in the buffer: 50 mM Tris-HCl (pH 7.5), 10 mM NaCl and 5 mM MgCl_2_ (10 μl) for 30 minutes and loaded on mica. Three-ten molar excess of linear DNA fragments or five-fifty molar excess of branched molecules was used for complex formation. Control samples were prepared by the same way, but without Dps. All samples were hold on mica for 5 minutes, washed twice with water for 30 seconds, dried, and the structure of complexes formed was analyzed by AFM Integra-Vita (NT-MDT, Russia) using cantilevers NSG03 with 10 nm tip curvature radius and 47–150 kHz resonance frequency. Measurements were done in semi-contact (tapping) mode. Images obtained were analyzed by Nova software (NT-MDT, Russia).

### Preparation of nicked DNA

To observe Dps complexes with DNA containing single-stranded breaks, 10 micrograms of plasmid pET28b were treated by Nt.BspD6I nickase [45]. The reaction mixture (20 μl) contained 10 mM Tris-HCl, pH 7.5, 10 mM MgCl_2_, 150 mM KCl, 1 mM DTT and 10 units of nickase or equal volume of Nt.BspD6I storage buffer (10 mM Tris-HCl, pH 7.5, 50 mM KCl, 0.1 mM EDTA, 1 mM DTT, 50% glycerol) for experimental or control sample, respectively. Digestion was allowed for 1 hour at 55°C. Plasmid DNA was immediately collected by phenol-chloroform extraction, precipitated by ethanol and dissolved in water. Melting curves were obtained for both plasmids, which confirmed nicking. Complexes with Dps in Dps:DNA molar ratio 5:1 or 10:1 were prepared as described above.

## Results

### Dps has different affinity to two fragments of *dps* promoter region

It is already known that deletion of *dps* altered the profile of proteins in the starved *E*.*coli* cells [14] and changed the profile of transcription in *S*.*enteritidis* [38] and in *E*.*coli* [39]. But this apparent regulatory effect may be mediated by interaction with regulatory proteins occupying their binding sites that are released in the *dps*-null mutant. In other words, the data obtained *in vivo* are suggestive, but not sufficient to give up the traditional point of view that Dps interacts with DNA without any specificity [1–3, 14–16]. We also previously found that two A/T-rich regions containing “*promoter island*” of *yeaI* and promoters of *dps* have higher affinity to Dps than two linear DNA fragments representing coding sequences and intergenic space located between convergent genes [46]. These experiments were performed *in vitro*, in the absence of any competition with regulatory proteins, which enhanced the possibility of selective interaction. Since transcription factors usually affect expression of their own genes, the *dps* regulatory region was selected as the most promising candidate to show the “specific” binding. Thus the 420 bp fragment covering regulatory region of the *dps* gene and interacting with the Dps protein was divided into two halves. One (259 bp in length) was obtained by PCR with primers *dps*_F2 and *dps*_R2 (Table 1) and included the main promoter of this gene—P_dps_ [47, 48]. Another one (214 bp long) contained distal promoter-like site P3, which demonstrated low transcriptional activity but was important for maximal expression of *dps* [49].

**Fig 1:**
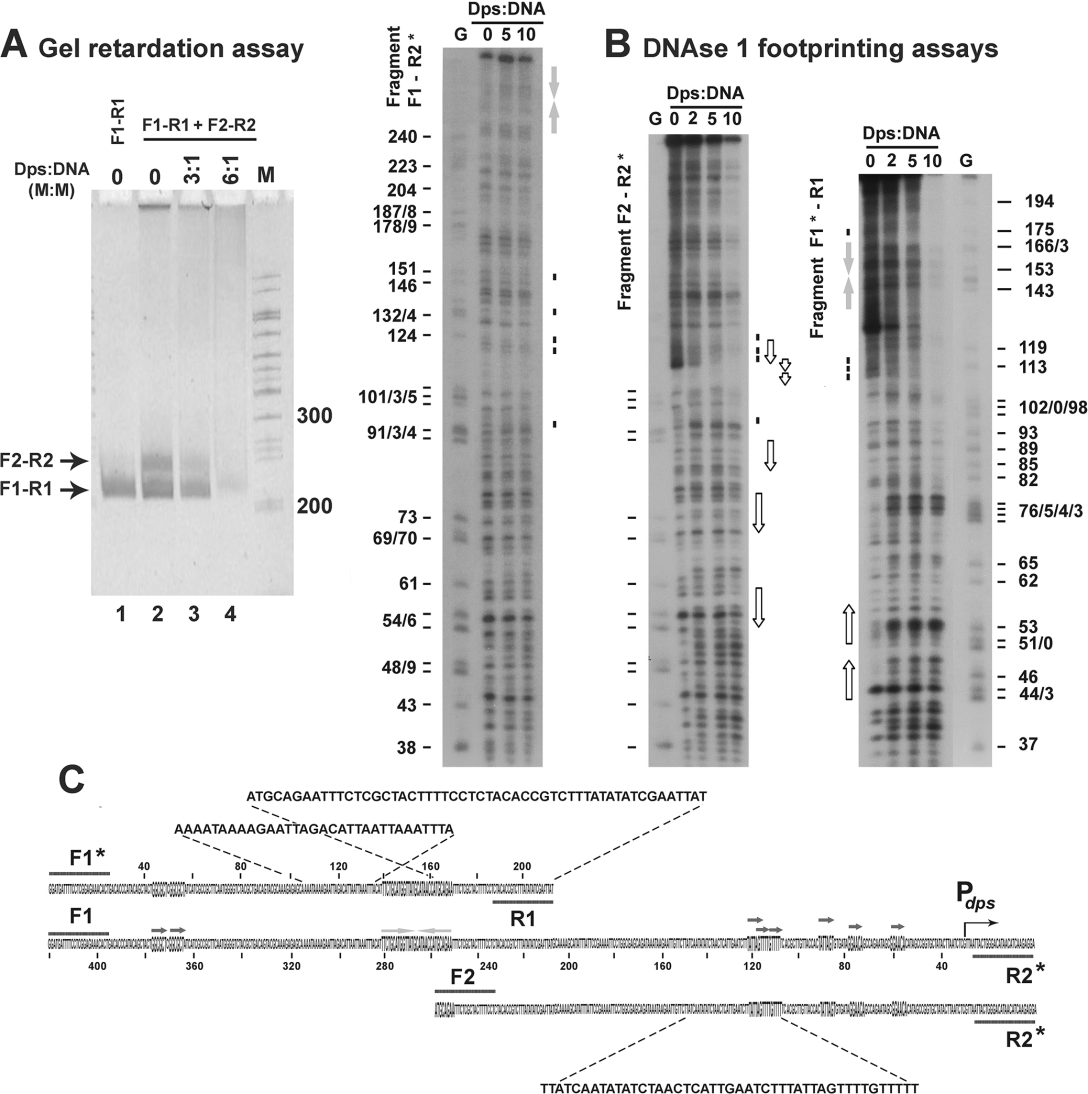
[A]: An example of electrophoretic mobility shift assays performed as described in Matherials and Methods. One pmol of the 214 bp-long DNA fragment amplified with primers *dps*_F1 and *dps*_R1 (designated as F1-R1) was loaded on the lane 1 alone as independent marker. Other samples contained two fragments (1 pmol each) and Dps as indicated above the photo. The gel was calibrated by marker ladder (M). **[B]** DNAse I footprinting assays performed for Dps complexes with F1-R1, F2-R2 and F1-R2 DNA fragments. ^32^P-labeled primers are indicated by asterisks. Gels were calibrated by the products of G-sequencing ladder. Positional marks show distance to ^32^P-labeled primers F1 or R2. Protected sites are dashed. Hyperreactive sites are not marked. **[C]**: Scheme illustrating relative disposition and structural organization of analyzed fragments. Direct and inverted repeats in their sequences are enhanced and additionally indicated by arrows. Corresponding bands in [B] are denoted by open and gray arrows for direct and inverted repeats, respectively. Bent arrow points transcription start site in P_*dps*_ promoter.

Since DNA-binding activity of Dps is usually accompanied by self-aggregation, and aggregated complexes do not enter the gel [1, 14, 19, 35, 39, 49], efficiency of binding was assessed on the basis of free DNA remained unbound. Using mixed assays, containing both halves of the regulatory region in one sample we observed that the fragment with functional promoter has higher affinity to Dps than the distal part of regulatory region (Fig 1A). Thus it became clear, that forming complexes with all DNA fragments tested so far [39], Dps can bind with certain selectivity to the promoter region of its own gene. We then tried to localize the Dps binding site in this region by DNAse I footprinting technique (Fig 1B). But revealing multiple hyperreactive sites and observing clearly protected R1- and F2-ends (scheme in Fig 1C) in both small fragments at large molar excess of Dps (10-fold), at 5-fold excess we found only several protected bands (~175 and ~113 bp downstream of primer *dps*_F1, and in the area 120–151 bp upstream of primer *dps*_R2). The latter was reproducibly observed in complexes with both short (F2—R2) and long (F1—R2) fragments, assuming some specificity in this binding. That is why at the next step we employed atomic force microscopy (AFM) to characterize the overall topology of the Dps-DNA complexes.

### Interacting with linear DNA fragments Dps usually binds the ends of double stranded molecules

**Fig 2:**
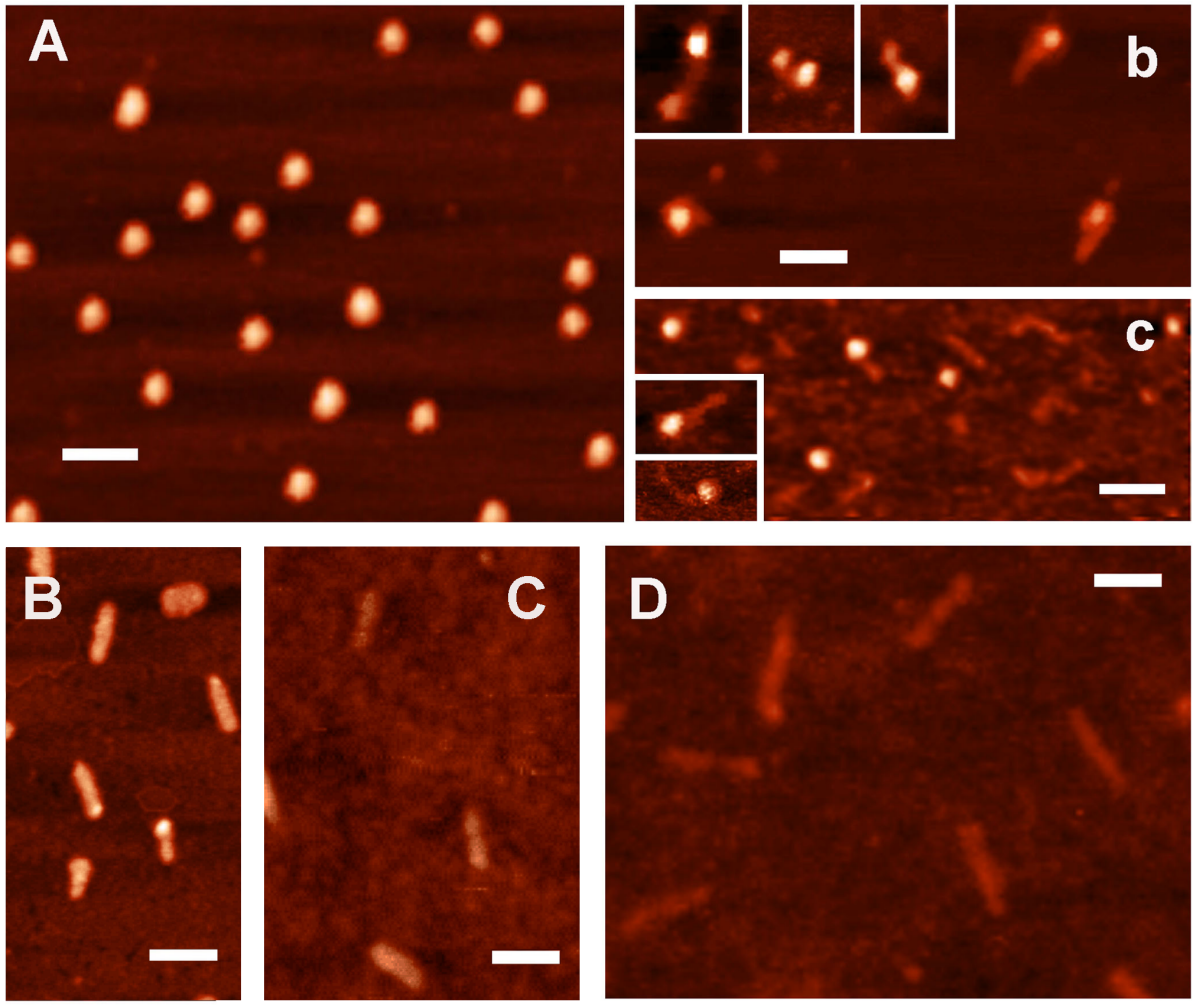
AFM images. **[A]**: Dps protein; **[B-D]**: DNA-fragments containing correspondingly complete regulatory region of gene *dps* (420 bp, primers *dps_*F1 and *dps_*R2), its proximal (259 bp, primers *dps_*F2 and *dps_*R2) and distal (214 bp, primers *dps_*F1 and *dps_*R1) part. Panels **b** and **c**: complexes formed by Dps with 214 bp **(b)** and 259 bp **(c)** DNA-fragments. White bar scales represent 100 nm.

Fig 2A demonstrates AFM images of purified Dps. Most particles observed were quasi-spherical in shape with height about 7 nm, which is in good agreement with the expected size [16]. Estimated vertical dimension of spread DNA molecules was 2 nm **(** Fig 2B–2D **)**, which also precisely fits to the expected value. However, the planar dimensions in AFM images depend on the finite size of cantilever tip (R = 10 nm in our case) and accuracy of their measurements depends on the size of the object. Thus, the apparent diameters of protein particles in planar projection were 27–32 nm, apparent width of DNA double helix—~21 nm, but lengths of 214, 259, and 420 bp DNA-fragments were 76, 93 and 146 nm, i.e. only slightly larger than expected values (73, 88 and 143 nm, respectively). The length of long DNA molecules can, therefore, be estimated quite accurately.

All protein samples appeared to be rather homogenous usually containing less than 20% of particles smaller than dodecamers. Surprisingly they were not contaminated by DNA fragments, which tight binding was *a priory* expected [14, 15], or aggregates (Fig 2A). However, the complex formation with DNA provoked severe aggregation. The removal of aggregates by washing of mica samples allowed registering individual complexes of Dps with both short DNA fragments used for electrophoretic mobility shift assays and footprinting (Fig 1). In both cases, we observed the interaction of protein particles with the ends of DNA molecules (Fig 2B and 2C). We have not seen any of ordered two-dimensional arrays previously registered by electron microscopy [14, 29–32, 34, 36]. But it was expected, since such complexes had never been observed by AFM [15, 18, 28] and we deliberately removed aggregates by extensive washing. We also found no hexameric rings embracing DNA [14] or convincing evidence of nucleosome-like DNA winding around the spherical Dps particles [36], which were expected in the framework of existing models. But we also did not find large number of the Dps molecules interacting with internal parts of linear DNA fragments. Even if such complexes may be specifically washed out with aggregated protein, it became clear that Dps can bind ends of double stranded DNA. Since fragments F1-R1 and F2-R2 overlap for 53 b.p. in their A/T-rich R1 and F1 ends, while two other regions with high A/T-content correspond to remaining two contact regions with Dps (Fig 1B and 1C), we presumed that higher affinity of this protein for the F2-R2 fragment (Fig 1A) is trivially explained by its lower thermodynamic stability (65 and 58% for F2-R2 and F1-R1 fragments, respectively).

### Y-shaped branched constructs are perfect targets for Dps

**Fig 3:**
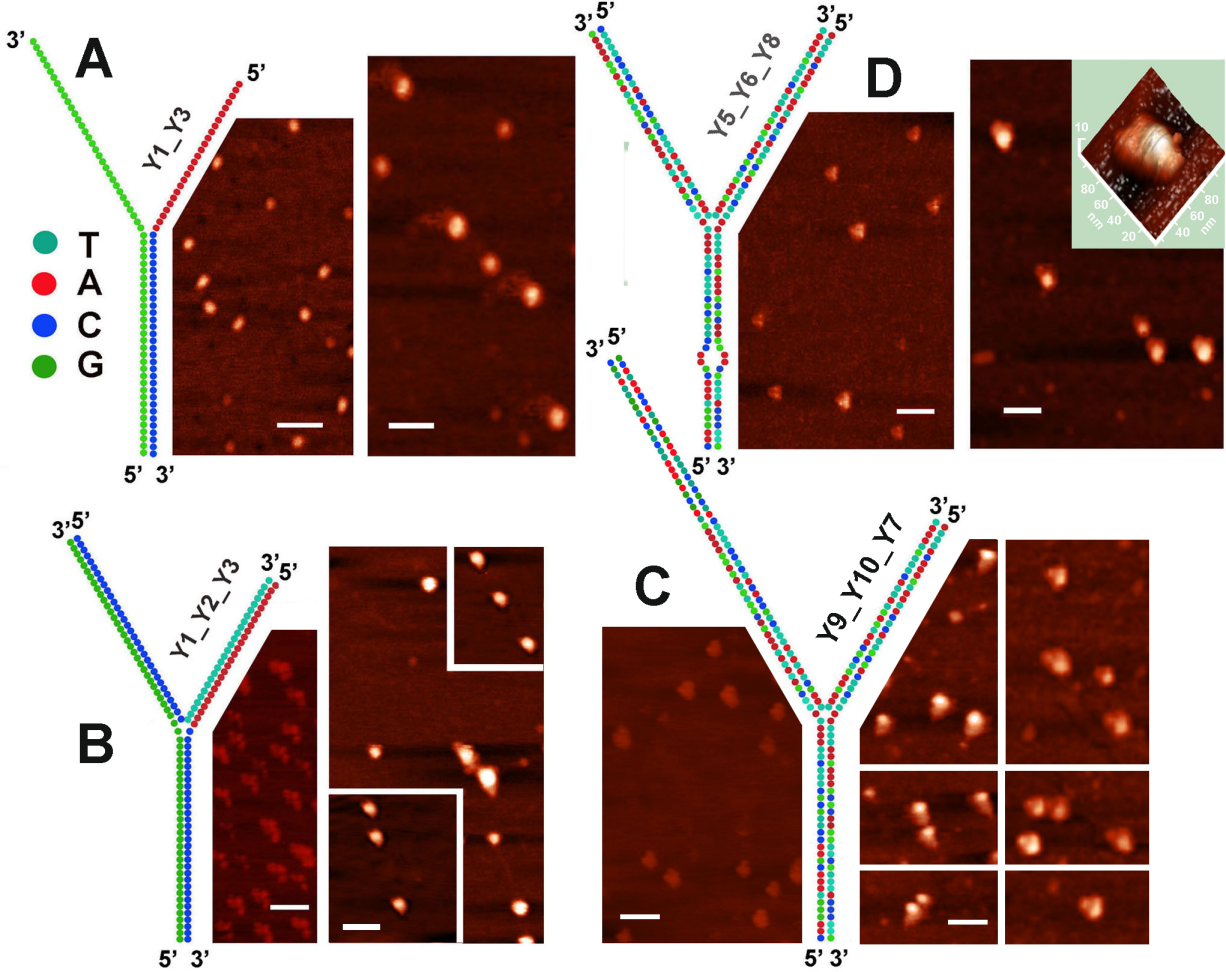
Complexes formed by Dps with four artificial branched constructs schematically drown on each section. The sequence of colored circles corresponds to the sequence of the oligonucleotides used (Table 1). Panels demonstrate AFM images obtained for free DNA samples and their complexes with Dps (left and right scans, respectively). Assembling of DNA constructs and complex formation were performed as described in Materials and Methods. Insert in the right panel of **Fig D** exemplifies the 3D image of complexes formed with Y5_Y6_Y8 triplex. Ends of all three branches are clearly visible. White bars represent 100 nm scales.

Two constructs were used to assess affinity of Dps to A/T-rich DNA. One of them was build of 2 synthetic oligonucleotides Y1 (57 n), and Y3 (64 n) (Table 1) as an artificial model of the DNA termini. It had a stable G/C-stem and two flexible single-stranded branches one of which was composed of adenines (Fig 3A). If Dps has enhanced affinity to the single-stranded DNA, we expected to find it in the complex with these single stranded branches, while G/C-stem could protrude from the complexes formed. The other construct was build of oligonucleotides Y1, Y2 and Y3 (Table 1) and contained three branches. Two long branches in this molecule had only G/C base pairs, while the short one contained only A/T pairs and might be the perfect target for the end-specific interaction (Fig 3B). In this case we expected to find Dps at the end of the short branch.

The left panel in Fig 3A exemplifies free DNA molecules assembled from primers Y1 and Y3. They all look like grains, but not as Y-shaped molecules. This may be due to the ability of guanines to quasi-complementary interaction with adenines and guanines. As a result, the apparent longitudinal size of most of the observed particles is about 25 nm, which is less than expected for a fully stretched duplex (32*0.34 + 32*0.59 = 29.8 nm). It is also possible that these binary complexes are intrinsically heterogeneous, since the 3’-terminal C of Y1 can bind not only the 5’-terminal G of Y3, but also any other guanine in its poly G sequence, thereby generating a mixture of different duplexes. However, all of them should contain at least a small portion of single-stranded DNA. Besides 25 nm—long grains, we also observed smaller particles with a size varying in the range of 13–20 nm, which may represent single oligonucleotides forming quadruplexes or other secondary structures.

Addition of Dps changed the structure of these particles. However, instead of detecting anticipated double-stranded helices extended from the binary complexes, we observed 2–4 disordered single-stranded DNA tails (Fig 3A). These tails had an apparent length of ~14–60 nm. If expected errors in planar projections were taken into account, they can be considered as two oligonucleotides with calculated length of 34 and 38 nm attached through their ends or inner parts to the N-termini (or surface) of Dps, or as single stranded branches of two Y-shaped molecules simultaneously interacting with one protein. In both cases, the maximal length of observed single-stranded tails is longer than single-stranded branches in correctly assembled duplex (15–20 nm), thus indicating that initial binding of Dps to the grain-like particles was strong enough to rearrange their structure, and to hold disordered molecules.

**Fig 4:**
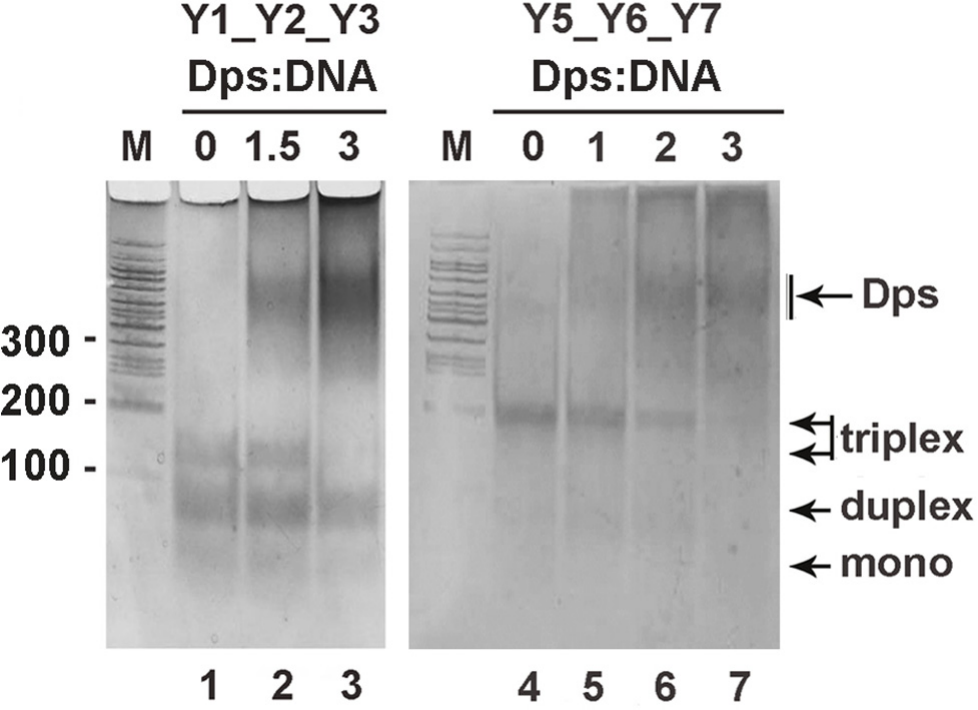
Electrophoretic mobility shift assays performed for Dps complexes with Y-shape constructs Y1_Y2_Y3 (drown in Fig 1B) and Y5_Y6_Y7 (similar to Y5_Y6_Y8 drown in Fig 1D but without single-stranded loop). The composition of the samples and the molar Dps:DNA ratio are indicated above photos. Branched DNA molecules were assembled from indicated oligonucleotides mixed in equal concentration (2–5 pmol each) and prepared as described in Materials and Methods. Without prior fractionation they were used for complex formation with Dps. The amount of Dps was chosen based on the assumption that all the oligonucleotides formed triplex, as in the case of Y5_Y6_Y7. Indicated molar ratios for Y1_Y2_Y3 are, therefore, overestimated. Gels were calibrated by marker DNA fragments (M) and stained by AgNO_*3*_ so as to visualize both DNA molecules and Dps.

Branched DNA molecules of Fig 3B were assembled from primers Y1, Y2 and Y3 (Table 1). They formed constructs resembling Y-shape (left panel), but were composed of an asymmetric V-shape module and a smaller domain associated with the main part. The size of this associate exactly matched to the small grains in Fig 3A, while the length of the V-shape module varied in the range of 24–30 nm (expected size in longest dimension was: 64*0.34 = 21.8 nm) and an average ratio between two sides was equal to 0.88 (expected value: 57/64 bp = 0.89). Thus, there is a possibility that self-assembling of observed particles proceeded through formation of complementary triplex associated with a duplex by non-canonical base pairing. Electrophoretic fractionation of these complexes really revealed two bands corresponding to triplex and duplex (Fig 4, lane 1). The shorter side of the V-shape module (Fig 3B) most probably corresponded to the A/T branch, while one G/C branch was conformationally hidden and may be in contact with the small associate. Orientation of the triplex in respect to this associate was random assuming their independent assembling from oligonucleotides. The mixture of assembled molecules also contained 10–20% of larger Y-shape particles (length: 53–62 nm, high: 2.6 nm of average), probably formed from triplexes and duplexes stacked due to quasi-complementary quadruplex-type interaction along the G-strand.

Both Y-shape structures interacted with Dps, but the protein in this case was found only in the central part of branched artificial constructs (right panel in Fig 3B). In stacked complexes it does not preclude the binding to the end of A/T- or G/C branch of the upper molecule. But small constructs with an apparent size of 24–30 nm also did not show the end-specific binding (inserts in the right panel of Fig 3B). As the size of these constructs was of the same value as apparent diameter of Dps in planar projection, they were almost completely covered by the protein, although the ends of all three branches were visible and identifiable.

To compare the affinity of Dps to the triplex Y1_Y2_Y3 and corresponding binary structures, we prepared a mixture of their self-assembled molecules and, without fractionation, subjected it for interaction with Dps (Fig 4 lanes 1–3). Three-fold molar excess of the protein was sufficient to bind all triplexes formed, while the majority of duplexes remained free. Based on these data, we assumed that Dps can bind the single stranded DNA and even melt the double helix (Fig 3A), but branched constructs that provide additional double-stranded platform for interaction with positively charged N-termini may be more preferred targets.

Next we found that the mode of interaction registered for Y1_2_3 construct (Fig 3B) was not a consequence of its specific primary structure, as the Y-shape molecules build of oligonucleotides with natural sequences Y5, Y6 and Y7 or Y8 formed the same type of complexes. The first half of Y5 (32 nucleotides) and the last half of Y6 were taken from the overlapping part of F1-R1 and F2-R2 fragments (Fig 1C). Self-assembling of these 96 bp constructs (exemplified for Y5_Y6_Y7 in Fig 4, lane 4) was much more efficient than in the case of Y1_2_3 triplex (Fig 4, lane 1). That is why only very small number of duplexes remained in the mixture. Nevertheless, in a 2-fold molar excess of the protein, when the number of triplexes significantly reduced, unbound duplexes were still detectable (lane 6). Thus, it is likely that among the ramified molecules with single- and double-stranded branches Dps mainly chooses the latter.

Two modifications were used to increase the quality of AFM images obtained for Dps complexes with branched DNA. First, we have added 26 nucleotides to the 5’-end of Y5 and to the 3’-end of Y6 (9 nm) so as to increase the length of the triplex (Table 1 and Fig 3C). The site protected by Dps against DNAse I, which is located 175 bp downstream of primer *dps*_F1 (Fig 1B and 1C), was herewith incorporated into the construct. As a result, we observed complexes with clearly visible one or two DNA branches (Fig 3C, two right columns). In the former case, Dps may be attached to the branching point, or to the end of one of short arms, while in the latter, the interaction with branching point seems more likely, but the third arm was poorly visible. Next we replaced TT dinucleotide in the internal part of Y_7 for AA (oligonucleotide Y8). As a result, new triplex Y5_Y6_Y8 possessed short single-stranded loop in one branch. This modification slightly shifted bound protein from the center (Fig 3D) showing the ends of all three branches (see 3D image in Fig 3D). Dps, therefore, binds 3-way junction and probably has certain specificity towards single-stranded or flexible regions in DNA. If so, binding of Dps to single stranded breaks in natural DNA might cause melting similar to that observed in Fig 3A. To check this possibility we analyzed complexes formed by Dps with native and nicked plasmid pET28b.

### Single-stranded breaks in natural DNA were not disordered by Dps

**Fig 5:**
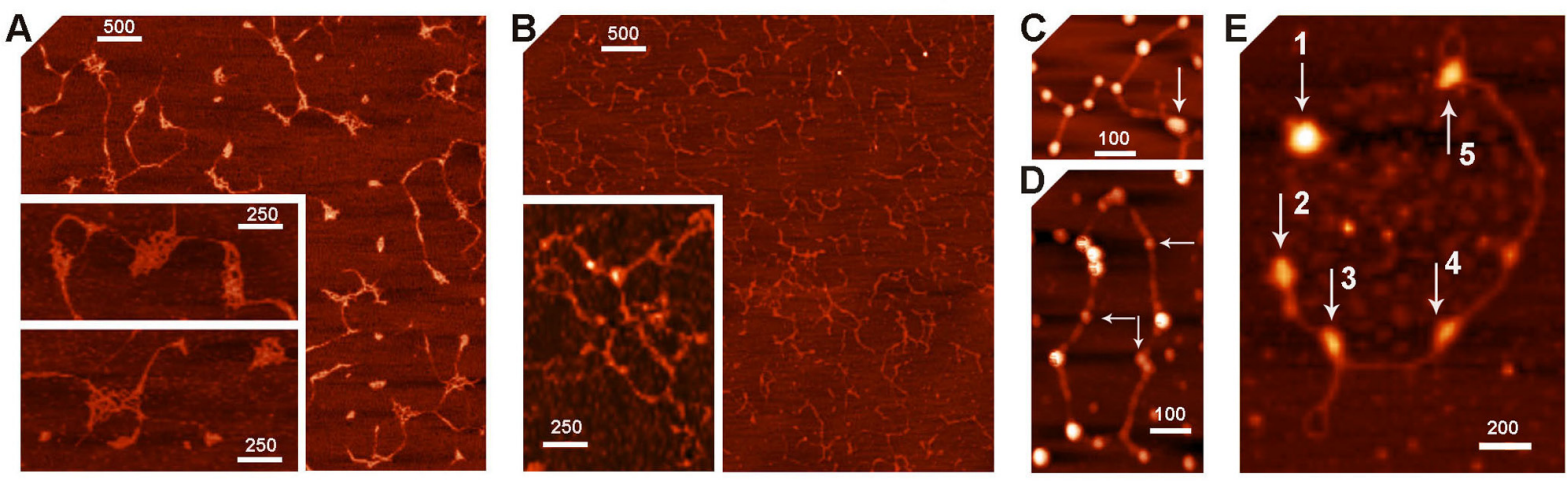
Examples of native (A, C) and nicked (B, D, E) plasmids pET28b in free state (A, B) and forming complexes with Dps **(C-E)**. White bars scale images (nm). Horizontal and vertical arrows in panels C-E point out complexes with lower and higher levels of oligomerization, respectively.

Purified plasmid pET28b looked like a native and strongly supercoiled molecule (Fig 5A). It was digested by the site-specific nickase Nt.BspD6I, which recognized nine GAGTC sequences and made single stranded breaks in the top strand four bases towards the 3’-end [50]. As a result, the plasmid became relaxed and even fragmented (Fig 5B) since several binding sites are closely located in this DNA. Complexes with Dps were formed in 5- and 10-fold molar excess of Dps dodecamers. The higher ratio ensured the presence of complexes formed with the cut and uncut DNA and allowed the detection of their difference, if any, while a lower ratio gave a chance to increase the portion of complexes formed with preferred sites. The density of bound Dps molecules in 10-fold protein excess was higher on nicked plasmid compared to that of the native one: 1 molecule per 117±12 nm versus 135±23 nm, respectively (the calculated length of the plasmid is 1917 nm). Even though the difference was not statistically significant, it was consistent with the expected contribution of nickes. However, binary complexes with dodecamers of Dps in both cases were very similar and thorough survey did not reveal single-stranded tails near bound protein (Fig 5C and 5D).

Both in native and nicked samples, we found approximately 15% of complexes formed with Dps particles smaller than dodecamers (indicated in Fig 5D by horizontal arrows). Dodecameric form, hence, is not absolutely required for the interaction with DNA. Fragmented DNAs in most cases have Dps molecules at least at one end of the double helix. End-specific interaction with Dps, thus, is not a peculiar property of short linear DNA fragments. Samples with nicked plasmid possessed twice more complexes formed with seemingly aggregated or somehow rearranged Dps particles (30 and 15%, respectively), which were usually embedded into DNA matrix (vertical arrows in Fig 5C–5E). Although their detailed structure requires special study, it is already clear that this mode of binding can cause significant conformational transitions in DNA, as supercoiled stem clearly visible near the complex 3 (Fig 5E) most probably can not be formed in the original plasmid, having gone through nickase treatment and sample preparation.

## Discussion

**Fig 6:**
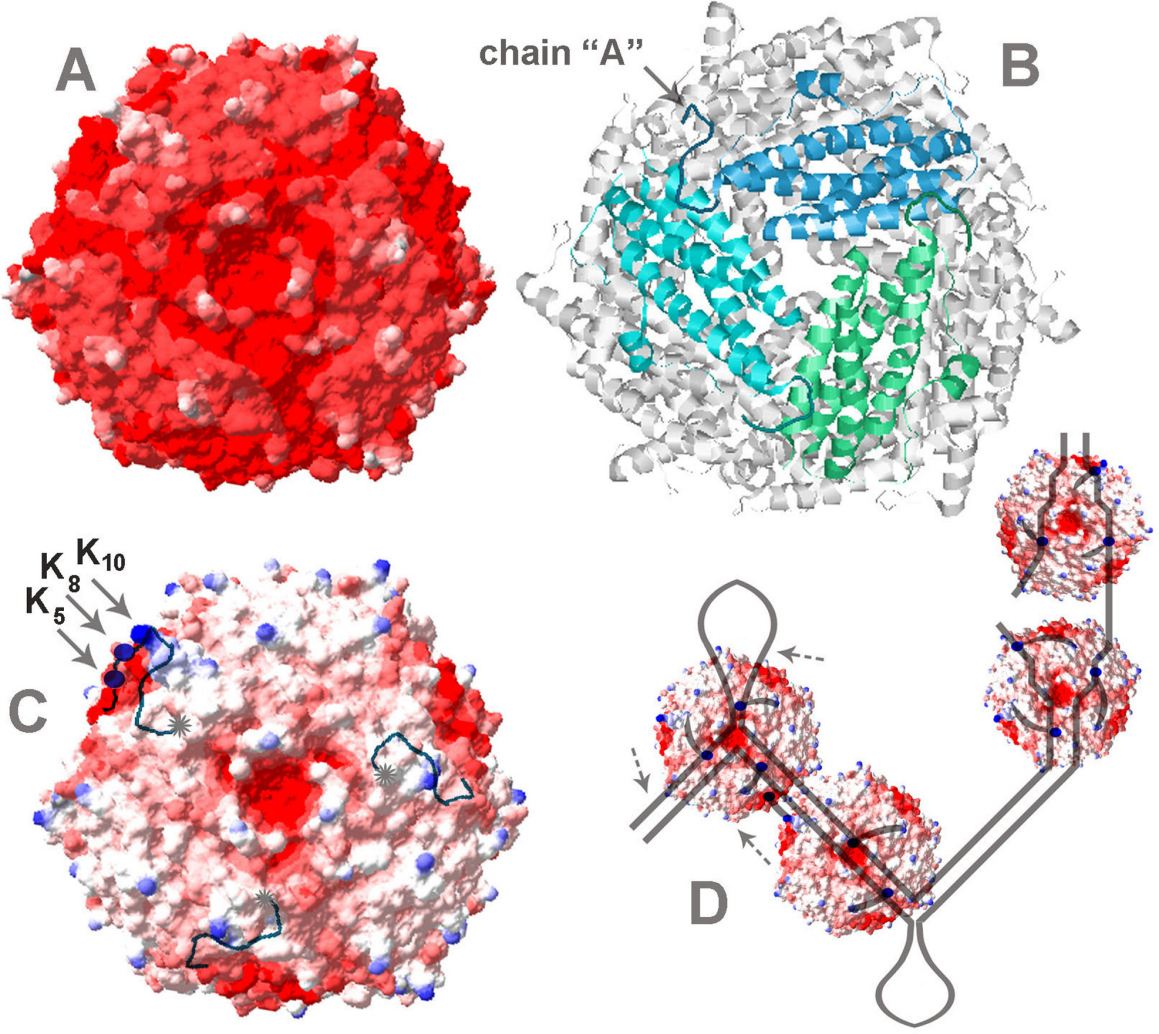
Crystal structure of Dps (PDB ID: 1DPS) was obtained with 1.6Å resolution by Grant et al [16]. Charge distribution on the surface of Dps was calculated using the Swiss-PdbViewer version 4.1.0 [52]. In panel **[A]** the threshold for Coulombic surface coloring was set at -12 for red (negative electrostatic potential), at -1.5 for white and at 0 for blue (positive potential). In panel **[C]** the scale was changed to: -12, -4.8 and 0, respectively. N-terminus of chain “A” was proportionally lengthened so as to show schematically the location of Lys_5_, and Lys_8_ (blue balls). N-termini of two other chains were lengthened up to position 9 (as in chain “A”). Gray asterisks in **[C]** mark ends of the flexible modules. Panel **[B]** shows the central pore and the disposition of subunits near the same vertex. Panel **[D]** schematically illustrates different modes of interaction, including binding to branched DNA of slipped loop structure (left), nicked DNA (two right particles), and straight DNA (second from the right). In the latter case, two N-termini are involved in DNA binding, while the third one of the same vertex can bind the vacant site of the negatively charged spot on the surface of the other Dps molecule. Even though such protein-protein contacts may be formed in solution, the presence of DNA must stabilize them and promote the protein aggregation. All molecular dimensions on the scheme are set in proportion to natural sizes.

Dps is the major protein of the bacterial nucleoid, condensing genome and protecting it from different damages during steady growth and under different stresses. That means that the interaction with DNA is a fundamental biological function of this molecule. The crucial role in binding belongs to lysine residues located at positions 5, 8, 10 and 18 of twelve N-terminal modules [15]. Their positive charge provides an ability to bind negatively charged DNA by the same way as histones in eukaryotic genomes do. However, in contrast to the histone octamer the spherical surface of Dps is charged negatively (Fig 6A). Thus, it is a mystery why evolution selected this protein for protective interaction with DNA.

Complexes with Dps were registered for all types of DNAs tested in this study, and two novel modes of binding (end-specific binding and interaction with branched DNA molecules) were observed. Since linear DNA fragments (Fig 2) or truncated DNAs (Fig 5) usually have Dps molecules at the end of double helix, we assume that end-specific interaction is more efficient than Dps binding to the internal parts of the DNA molecules. However artificial triplexes overtook duplexes in complex formation with Dps (Fig 4, lanes 1–3) apparently attaching it to the internal part of the construct (Fig 3). We explained this preference by the presence of a binding site for additional N-terminus in both new targets. Since in the structure of dodecamer DNA-binding modules of monomers are grouped in triads (Fig 6B), the three-way junction may be particularly advantageous for complex formation, the same as curved and uncoiled DNAs, including partly melted ends of the DNA double helix and single-stranded breaks, while interaction with straight DNA can be limited by only two contacts with N-termini. If single-stranded breaks are the targets for Dps, then already known ability of this protein to reduce their number in response to oxidative stress [51] may be mediated not only by physical protection of DNA, but also by its active participation in binding to such defects.

Although the first 21 amino acid residues in each Dps subunit are unstructured, 13 of them can be tracked in X-ray structure [16] using as a prototype the polypeptide chain “A” (indicated in Fig 6B) [16]. Thus, it is clear that the unstructured N-termini are quite long and can be engaged in electrostatic interactions with both negatively charged DNA and negatively charged protein surface. I.e. they may adhere to the protein by electrostatic forces as it was observed for Dps of *A*. *tumefaciens* [25] and may be undetectable in the crystal structures either because they are flexible, as it is commonly believed, or because their contact sites are randomly distributed over the entire available surface. That is why the ability of Dps to aggregate and form oligomeric structures is its intrinsic property. But this ability is greatly stimulated by the presence of DNA and binary Dps-DNA complexes were almost never visible in gel-shift assays [1, 14, 19, 34, 39, 49]. It is well known that the main players in this process are the same N-terminal lysines that bind DNA [15]. Thus, it was suggested that random binding of the Dps molecules to DNA limited their mobility in space thereby increasing the protein concentration on the genome and favoring protein-protein interactions [15, 18, 28]. This model explains very well the formation of large aggregates often observed by AFM, but can not explain formation of highly ordered two dimensional honeycomb-like arrays [14, 29] and low ability of free Dps to aggregate even in a very high concentration (see Fig 2A, for instance). Our data, emphasizing the importance of the 3-fold symmetry allowed us to offer another explanation for the strong dependence of the aggregation process on the presence of DNA. We assume that in the absence of DNA N-termini of Dps are not completely free. Instead, they are randomly immobilized on a negatively charged protein surface. Three highly charged spots were revealed by surface analysis using the Swiss-PdbViewer [52] (Fig 6C). They are located at a convenient distance for each N-terminus, providing an ideal platform for the binding, which reduces the probability of random external electrostatic contacts. In the presence of DNA, one, two or three DNA-binding modules of each vertex can leave their binding sites, releasing them for contacts with N-termini of other Dps molecules, if they are located in an accessible distance. Two left particles in Fig 6D exemplify this situation. I.e., from our point of view, the presence of DNA stimulates protein-protein connections by the redistribution of electrostatic contacts. Those of them, which stabilized the integrity of the dodecamer without DNA, cause aggregation in new conditions.

The ability of Dps to recognize branched double stranded DNA may be even of greater biological significance. Only one system is currently known in *E*.*coli*, which recognizes and processes such branched structures as Holliday junctions. These four-way junctions are formed during recombination and DNA repair and are resolved by the system composed of three proteins RuvA, RuvB and RuvC, where octameric helicase RuvA is specifically “sculptured” for interaction with cruciform DNA [53]. Three-way junctions may be formed at any DNA segment containing at least two direct repeats, where two types of slipped loop structures (SLS, exemplified in Fig 6D) can be formed [54]. In bacterial genomes, there are thousands of such places including clusters of regularly interspaced short palindromic repeats (CRISPR), belonging to the bacterial “immune” system and tandems of transcription factor binding sites. In the promoter region of the *dps* gene, for instance, there are four pairs of short direct repeats, two of which overlap with the primary contact site for Dps (Fig 1B and 1C). The structural state of genomic regions with tandem repeats should be under the special control of cellular regulatory systems. But the system evolutionarily adapted for this function is not yet known, and bacterial Dps can be suggested as a candidate for this function. Its affinity to 3-way junction and ability to cause conformational changes in DNA (Figs 1B, 3A and 5E) are weighty arguments in favor for such a possibility. Though, the ability of Dps to aggregate, which is basically important for the genome condensation in stress conditions, can interfere with the delicate functioning, required to control structural landscape in the active genome.

There is one aspect that also deserves some attention: if the DNA is bound by three N-termini of one vertex, which is supposed to be the strongest mode of interaction, the central pore of the protein globule leading into its inner cavity (Fig 6C), comes to the closest vicinity with the genetic material. Even a small leak of toxic iron ions from this pore may be destructive for the integrity of the genome. Thus, it is likely that protecting DNA from various damaging agents and removing toxic iron ions from the genomic environment, Dps might possibly be also involved in a structure-specific destruction of nucleic acids.

In any case, our data showed that the purified Dps of *E*.*coli* is assembled into stable dodecameric particles with some contribution of smaller oligomers. Interacting with DNA the dodecameric form of Dps demonstrated certain end-specificity and high affinity to three-way junction in artificial DNA molecules. As Dps binding to DNA is mainly driven by electrostatic interactions, there is no reason to exclude that Dps can also form complexes with RNA affecting their functional properties or stability, especially as it has already been reported that “coral-reef structutres” formed by Dps2 of *M*. *smegmatis* can be destroed by RNAse A [55]. The repertoire of multifunctional protein Dps may, therefore, be even broader than currently anticipated.
